# Reaching consensus on an analgesia protocol for paediatric burn patients in a resource-scarce South African community

**DOI:** 10.4102/safp.v63i1.5193

**Published:** 2021-02-23

**Authors:** Shelley L. Wall, Nikki L. Allorto, Verusia Chetty

**Affiliations:** 1 Pietermaritzburg Burn Service, Pietermaritzburg Metropolitan Department of Surgery, Nelson R. Mandela School of Medicine, University of KwaZulu-Natal, Durban, South Africa; 2 Developing Research, Innovation, Localization and Leadership (DRILL), College of Health Sciences, University of KwaZulu-Natal, Durban, South Africa; 3 Discipline of Physiotherapy, School of Health Sciences, University of KwaZulu-Natal, Durban, South Africa

**Keywords:** analgesia protocol, low- and middle-income countries, LMIC’s, burns, paediatrics, resource-limited

## Abstract

**Background:**

Despite the exceptional burden of burns in low- and middle-income countries (LMIC) and the importance of adequate analgesia in burn care, there is a lack of analgesia protocol developed in resource-scarce settings. This necessitates the development of an analgesia protocol applicable to the resource-scarce setting. This study presents the findings of a modified Delphi study aimed at achieving consensus by a panel of experts in the management of burn injuries from low- and middle-income settings across Africa.

**Methods:**

A two-round Delphi survey was conducted to achieve consensus on an analgesia protocol for paediatric burn patients for a resource-limited setting. The Delphi panel consisted of nine experts with experience in management of burn injuries in low-income settings.

**Results:**

Consensus was determined by an a priori threshold of 80% of agreement for a drug to be included in the analgesia protocol. There was a largely overarching agreement with regard to the background analgesia protocol and strong agreement regarding the use of an initial dose of ketamine and midazolam for procedural sedation.

**Conclusion:**

A modified Delphi method was used to obtain expert consensus for a recently adopted analgesia protocol for burn-injured children in a resource-limited setting, with experts in the management of burn injuries in low- and middle-income settings. The expert consensus leads to the rigour and robustness of the protocol. Delphi methods are exceptionally valuable in healthcare research and the aim of such studies is to find converging expert opinions.

## Introduction

The burden of burn injuries on sub-Saharan countries, especially amongst children, is huge. Of all the children in the under – five age group in this region, it is estimated that between 300 000 and 17.5 million children sustain burn injuries annually.^1,2^ In the medical approach to care, it has long been recognised that inadequate pain control can have adverse physiological and emotional sequelae. Despite this, pain control remains inadequate, not only in the sub-Saharan region but across the globe.^2,3,4,5^ Adequate analgesia in burns is essential, but it is often difficult to achieve. Additionally, burn pain is dynamic and needs constant reassessment by medical practitioners.

There is a lack of analgesia protocols which have developed in resource-scarce settings despite the exceptional burden of burns in low- and middle-income countries (LMIC) and the importance of adequate analgesia in burn care. Whilst analgesia protocols have been published, these are predominantly developed in high-income countries (HICs) and may not be applicable in LMICs with their limited availability of medication and monitoring equipment. Worldwide, there is uneven distribution of resources for the administration of adequate analgesia to children for painful procedures.^6^ In many regions, such as sub-Saharan Africa, these resources are exceptionally scarce.^6^ This necessitates the development of an analgesia protocol which is applicable to the resource-scarce setting.

Previous studies show lack of knowledge and poor clinical practice in the area of analgesia and burns in our setting.^7,8^ There is an abundance of knowledge and practices published on this topic but tend to be very generalised.^9,10,11^ Burns in our setting are managed by interns to senior medical officers both in our institution and those that refer to us. They have varying experience with pain management and it is most commonly lies beyond their area of expertise. Protocols can be a practical starting point for doctors with only general skills. Not providing a protocol leads to varying degrees of care that is at risk of falling below an acceptable standard.^12^ The protocol was thus developed by surgeons and anaesthetists with experience in burn and pain management to offer assistance to those with less expertise. It is highly unlikely that this problem is unique to us.

This study presents an expert consensus on an analgesia protocol that has recently been adopted by our service. The analgesia protocol presented for consensus, aims to provide safe analgesia strategies to improve pain control for paediatric burn patients for use at district, regional and tertiary hospitals across KwaZulu-Natal (KZN). This study presents the findings of a modified Delphi study aimed at achieving consensus by a panel of experts on pain management for burn injuries from low- and middle-income settings which could be adapted for application in other LMIC’s across Africa. Expert opinions were explored to strengthen the proposed analgesia protocol for paediatric burn patients in KZN.

The basis of the initial questionnaire for this Delphi study was the work previously done in analgesia in paediatric burn patients conducted in the same setting.^7,13,14^ These previous studies highlighted the lack of knowledge of doctors with regard to analgesia options and doses for children with burn injuries. Although there are challenges with the implementation of protocols, analgesia protocols remain invaluable to aid healthcare professionals with little experience in the management of burn-injured children.^7^ They also emphasised the complications of inadequate analgesia, reiterating how imperative adequate analgesia is from the outset in the management of these patients.^13^ The provision of analgesia to burn-injured children can be divided into background analgesia and procedural analgesia. Sections dedicated to each of these were included in the protocol and included in the Delphi survey.^15^

## Setting

The Pietermaritzburg Burn Service (PBS) is managed by two burns surgeons and operates across the regional (Edendale Hospital) and tertiary (Greys Hospital) hospitals in Pietermaritzburg. The two burns surgeons manage the patients admitted to the 40 dedicated burns beds across the metropolitan but also offer support to all the regional hospitals who refer to the PBS. The PBS provides support to 19 district hospitals in the western third of KZN Province. The doctors in these hospitals who refer to the PBS have access to the burns surgeon on call 24-h a day for advice on the management of all burn-injured patients. Patients in western KZN are managed according to ‘The PMB Way Burns Protocols’ and the aim is that all burns patients are discussed with or seen by one of the two burns surgeons.

## Methods

A modified Delphi technique was used to engage an expert panel of doctors managing burn injuries in low- and middle-income settings, in order to gain consensus on an analgesia protocol for paediatric burn patients applicable for KZN. This was conducted between October 2019 and April 2020.

In accordance with the tenets of the Delphi approach, methodological rigour was maintained through the consensus of expert opinion from medical practitioners who are experienced in the management of burn-injured children in low-resource settings, albeit dispersed geographically across the African continent. This is founded on the belief that the collective views of a group of experts are preferable to those of an individual.^16^ Anonymity, iteration, controlled feedback and group response further enhanced rigour.^16,17^ Additionally, researchers used purposive sampling, an emergent design and structured communication to satisfy the underpinnings of a consensus survey.^16^

The lead researcher distributed invitations, which included the study information letter and the ethical approval, as well as the first-round questionnaire, in person to the experts at a burns congress where all the experts had converged. The first-round questionnaires were returned and a research assistant de-identified the questionnaires to ensure anonymity. The second-round questionnaire was conducted using a Google form. A link to the Google form was e-mailed to the panellists. The form was anonymised prior to analysis. Through iteration and by communicating results from the previous round to the expert panellists, stability of the feedback was established.^18^

## Panel recruitment

There is a great shortage of experts in the management of burn-injured patients in Africa, and South Africa is no exception to this problem.^19,20^ Because of a lack of experts in certain healthcare fields in LMICs, studies in these healthcare contexts lend themselves to the recruitment of a smaller number of panellists.^16^ In keeping with the Delphi healthcare research approaches, 10 experts were identified and invited to participate.^17^

A panel of experts in the management of burn injuries in low-income settings was selected. Ten experts were identified through burn organisations known to work in low- and middle-income settings. The potential participants included general surgeons, plastic surgeons, paediatric surgeons and anaesthetists. The criteria for being included in the expert panel were medical doctors, having 10 or more years’ experience in the management of burn-injured patients in a resource-limited setting, and/or with published research in the field of paediatric burn injuries. One of the identified experts, after agreeing to participate in the study, did not return the first-round questionnaire, and on completing the first-round questionnaire, another expert did not respond to further e-mails regarding round two. Round one, therefore, consisted of nine experts and round two consisted of eight experts. Unfortunately because of the limited number of specialists who manage burns on a regular basis in Africa, there are a very limited number of people who meet the criteria as an expert in the field. The majority of burns are managed at the primary healthcare or district healthcare level by generalists, who only occasionally manage burns patients, often with limited training in the management of such patients.^20,21^ As a result, we were not able to replace the panellist who accepted the questionnaire but did not return it.

## Overview of the Delphi process

**FIGURE 1:**
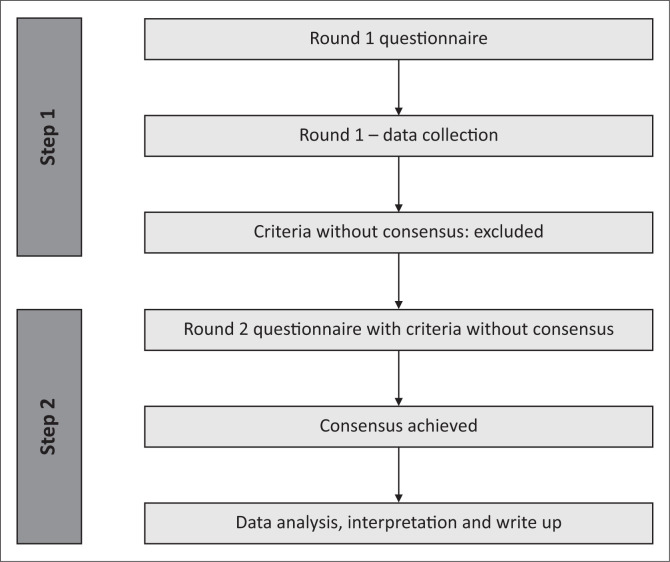
Overview of the Delphi process.

Consensus was achieved following a two-round modified Delphi survey. An overview of the Delphi process used for this study is depicted in Figure 1.

*Step 1* involved an analgesia protocol that we had recently adopted and was framed in a q uestionnaire which included a four-point ordinal scale to rate the specific drug of the protocol as: (1) *Essential:* the drug must definitely be included in the protocol; (2) *Useful:* the drug can be included in the protocol; (3) *Unnecessary*: the drug must definitely be excluded from the protocol and (4) *Unsure:* unsure about this drug.^22,23,24^ The protocol, and therefore the questionnaire, was divided into two sections: ‘Background analgesia and sedation’ and ‘Procedural analgesia and sedation’.^24^ All drugs are shown in Table 1. The first-round questionnaires were then distributed to the panellists, together with the study information letter and informed consent. The data from the first round were then analysed and used for the generation of the second-round questionnaire. The data from round one was collated and analysed using Excel version 16.35. An a priori threshold of 80% agreement determined consensus, using frequency distributions on *essential* and *useful* responses collaboratively on the four-point scale.^24^

**TABLE 1:** A summary of the consensus for each of the drug items across the two rounds.

Variable	Round 1 (*n* = 9)				Round 2 (*n* = 8)			
	Essential/useful (of those who responded)		Unnecessary/unsure (of those who responded)		Essential/useful		Unnecessary	
	*n*	%	*n*	%	*n*	%	*n*	%
**Background analgesia and sedation**								
**Mandatory**								
Paracetamol 15 mg/kg 6-hourly	9/9	100.0	-	0.0	-	-	-	-
**Mandatory**								
Tilidine 1 mg/kg 6-hourly	4/8	50.0	4/8	50.0	6/8	75.0	2/8	25.0
**Add if pain not controlled and for donor site pain**								
Ibuprofen 10 mg/kg 8-hourly	8/8	100.0	-	0.0	-	-	-	-
Consider contraindications of Ibuprofen: Curling’s ulcer, acute kidney injury and comorbidities	4/4	100.0	-	0.0	9/9	100.0	-	0.0
**Consider if > 15% TBSA**								
Morphine syrup: Start at 0.2 mg/kg 6-hourly. Increase frequency up to 2-hourly then increase dose by 25%. Consider an infusion.	9/9	100.0	-	0.0	-	-	-	-
**Add if pain not controlled or neuropathic pain**								
Clonidine 25 mcg 8- hourly. Increase to maximum 50 mcg 8-hourly	8/9	88.9	1/9	11.1	-	-	-	-
**For neuropathic pain and or severe itch**								
Pregabalin start at 25 mg 12-hourly. Increase in 25 mg increments to max 75 mg 12-hourly	6/6	100.0	-	0.0	8/8	100.0	-	0.0
Gabapentin 10 mg/kg 8-hourly. Increase in increments of 100 mg/dose up to 600 mg 8-hourly	6/6	100.0	-	0.0	8/8	100.0	-	0.0
**If itch and no Pregaba/Gabapentin**								
Allergex 0.1 mg/kg. Start 12-hourly, can be increased to 8-hourly	7/8	87.5	1/8	12.5	7/8	87.5	1/8	12.5
**For ICU patients/large TBSA burns** **(MORPHINE mixed as 1 mg/ml solution, i.e. 10 mg in 10 mL or 50 mg in 50 mL)**								
Morphine IVI 0.1 mg/kg loading dose, then 0.1 mg/kg/h infusion Increase to effect, reload and increase rate by 0.05 mg/kg	8/8	100.0	-	0.0	-	-	-	-
**For PTSD or anxiety or opioid withdrawal**								
Valium 2.5 mg nocte, titrate to effect. Can be increased to 8-hourly	5/7	71.4	2/7	28.6	7/8	87.5	1/8	12.5
**Procedural medication**								
**IV access/ICU/high care**								
Ketamine 1 mg/kg IVI titrations. Quick onset, quick offset	9/9	100.0	-	0.0	-	-	-	-
**Ward dose 1**								
Ketamine 5 mg/kg per os Midazolam 0.25 mg/kg per os mixed together. 20–30 mins to work	9/9	100.0	-	0.0	-	-	-	-
**Ward dose 2 (for pain score > 3)**								
Ketamine. Half the previous ketamine dose given IMI. 5–10 mins onset. NO midazolam	6/9	66.7	3/9	33.3	7/8	87.5	1/8	12.5
**Ward dose 3 (for pain score > 3)**								
Ketamine half the previous ketamine dose IMI	6/8	75.0	2/8	25.0	8/8	100.0	-	0.0
The final total dose of ketamine given at the procedure must be written as the script for the following dressing change. Do not leave the inadequate dose as the prescription	1/1	100.0	-	0.0	8/8	100.0	-	0.0
**Clinic**								
Ketamine 5 mg/kg IMI OR Methoxyflurane 0.5 mL inhaled	6/7	85.7	1/7	14.3	7/8	87.5	1/8	12.5
**Emergency department**								
Ketamine 5 mg/kg IMI	9/9	100.0	-	0.0	-	-	-	-

TBSA, total body surface area; PTSD, post-traumatic stress disorder; IMI, intra-muscular injection; per os, per mouth.

*Step 2* involved the development of the second-round questionnaire using only the questions where consensus was not obtained in the first round. A similar ordinal scale was used; however, this was reduced to a three-point ordinal scale: (1) *Essential;* (2) *Useful and* (3) *Unnecessary.*^25,26^ This questionnaire was distributed to the panellists via an e-mail link to a Google form which the panellists completed online. Round-two questionnaires were returned over 4 weeks. Unfortunately, only eight of the panellists replied and despite numerous e-mails, the last panellist did not respond. There was a high level of consensus in round two, which is shown in Table 1. The Delphi study was concluded once the objective of the study had been met and consensus had been achieved with regard to the content of the analgesia protocol.

### Ethical consideration

This study was granted ethical clearance by the Biomedical Research and Ethics Committee of the University of KwaZulu-Natal with clearance number: BE594/18. The participants signed an informed consent letter after reading a study information document. Anonymity was maintained throughout the study, as described above. No incentives were offered for participation. This article followed all ethical standards for research without direct contact with human or animal subjects.

## Results

The modified Delphi survey was conducted in two rounds. The first round included nine panellists. In the second round, one of the initial nine panellists opted out of the study. The demographics of the panellists are summarized in Table 2.

**TABLE 2:** Demographics.

Variable	Round 1 (*n* = 9)	Round 2 (*n* = 8)
**Gender**		
Male	100.0%	100.0%
**Age in years**		
31–40	11.1%	12.50%
41–50	22.2%	12.50%
51–60	44.4%	50%
> 60	22.2%	25%
**Profession**		
Plastic surgeon	44.4%	37.50%
General surgeon	33.3%	37.50%
Anaesthetist	11.1%	12.50%
Paediatric surgeon	11.1%	12.50%
**Years’ experience in the health profession**		
11–15	11.1%	12.50%
16–20	11.1%	12.50%
> 20	77.8%	75%
**Years’ experience in burns**		
6–10	11.1%	12.50%
11–15	11.1%	12.50%
16–20	22.2%	12.50%
> 20	55.6%	62.50%

The first round of questionnaires yielded agreement on the majority of the drugs in the protocol. There was a high level of consensus (more than 87% of the panellists) for nine of the 18 questions. We will discuss the findings in terms of ‘Background analgesia and sedation’ and ‘Procedural analgesia and sedation’. The consensus for each of the drug items across the two rounds is presented in Table 1.

### Background analgesia and sedation

There was overarching agreement with regards to the use of paracetamol, ibuprofen, morphine and clonidine in a stepwise manner in the analgesia protocol. Half of the panellists were in agreement with regards to tilidine (Valeron^®^) and the other half had no experience with it as it was not available in their settings, as was indicated in their additional comments. In the second round of the Delphi survey, there was 75% agreement, but consensus was still not achieved. The majority of the panel (87.5%) agreed that Allergex^®^ (chlorpheniramine) should be included in the protocol for the treatment of itch.

### Procedural analgesia

There was strong agreement regarding the use of an initial dose of ketamine and midazolam for procedural sedation, with all of the panellists believing this was essential. The majority (at least 87.5%) also felt that additional doses of ketamine were required should adequate analgesia not have been achieved with the initial doses.

## Discussion

Burn injuries, and the management thereof, are fraught with not only the pain associated with the burn wound, but also with painful procedures required to ensure recovery. All children with a burn injury will experience pain during their treatment and recovery.^27^ All over the world, the management of burn pain remains inadequate, despite extensive evidence of the negative physiological and psychological impact of pain on children.^27,28^ Poor pain control may result in delayed wound healing and long-term sensory problems, as well as debilitating long-term psychological conditions and chronic regional pain syndromes.^27,29,30^

The management of pain is essential in burn-injured children. Worldwide, resources for the delivery of services, such as analgesia and anaesthesia during surgery and painful procedures for both children and adults, are unevenly distributed; in sub-Saharan Africa, these resources are exceptionally scarce.^6^ Due to the fact that health-provider training in paediatric anaesthesia and analgesia is especially uncommon in many low-income countries, the lack of healthcare providers to deliver anaesthesia and analgesia to children is even more significant than for adult patients.^31,32,33^ Whilst there are analgesia protocols available for the management of burn-injured children, the majority of these are either for HICs or are developed for LMICs by HICs. These protocols often do not take cognisance of the lack of monitoring or drug restrictions in a resource-limited environment.

Published protocols remain vague which still poses a problem for healthcare providers who are unsure how to manage pain for burn-injured patients. We aimed to provide a protocol with specific recommendations in terms of doses, when and how to titrate. With this in mind, the researchers purposed to develop an analgesia protocol which was a collaborative effort by a team of surgeons and anaesthetists with both clinical experience with burn dressings and extensive knowledge regarding monitoring capabilities, and the lack thereof, in resource-limited settings, as well as drug availability. Using a Delphi survey, the researchers sought expert consensus from doctors from resource-limited settings to develop an analgesia protocol for burn-injured children in resource-limited settings.

The expert panel agreed that provision needed to be made for both background analgesia and procedural analgesia. Whilst this may seem intuitive, another factor dividing the developed and the developing world is analgesia, especially in the paediatric population.^34^ In the face of limited resources, the provision of pain relief for burns is a challenge because of a limited spectrum of analgesics, inadequately trained staff and a lack of monitoring equipment.^34^ In round 1 of the modified Delphi survey, there were numerous drug items with no responses. The researchers deduced that insufficient access to certain drugs resulted in failure to answer questions about those drugs but there is no evidence to support this deduction.

In terms of background analgesia, there was consensus amongst the expert panellists regarding the inclusion of paracetamol and morphine in the analgesia protocol for a resource-limited setting. There was no consensus on the use of tilidine (Valeron^®^). The reason for this lack of consensus was its unavailability in many resource-limited settings, and therefore the researchers have removed it from the analgesia protocol. However, when tilidine is available, researchers would still advocate its use. The use of ibuprofen for background pain was unanimously agreed on.

Burn pain is dynamic in nature and this is, in part, related to the fact that the hypermetabolic response to burn injuries result in the altered metabolism of analgesic drugs.^35^ In burn-injured patients, there is an inevitable complex interaction between pain and anxiety, and this also contributes to the dynamic nature of burn pain.^36^ The dynamics of burn pain necessitates constant reassessment of the analgesia plan for these patients. If the first three tiers of analgesia, namely paracetamol, ibuprofen and morphine are ineffective to achieve adequate analgesia, clonidine is another useful drug in the armamentarium against pain. There was consensus amongst the experts that clonidine is an essential part of the analgesia protocol. Clonidine has opioid-sparing effects which are useful in addressing tachyphylaxis that burns patients experience.^27^ It has been demonstrated in the literature that the benefits of clonidine are not limited to improved analgesia and the morphine-sparing effects, but clonidine also reduces sympathetic overactivity associated with burns.^37^

There was consensus regarding the inclusion of pregabalin and gabapentin for burn pruritus. Post-burn itch is a distressing syndrome, the severity of which is variable.^38^ The severity of burn pruritus is usually most severe immediately after wound closure.^39^ In paediatric burn-injured patients, post-burn pruritus is highly prevalent.^40^ Whilst burn itch was historically managed by emollient massage and antihistamines, both gabapentin and pregabalin have been shown to be very effective to treat it. The expert panellists acknowledge these drugs as being essential for the analgesia protocol.

Dressing changes are an unavoidable part of burn care. There is a limit on theatre time in LMICs. As a result, it is not possible for burn-injured children to have their dressing changes exclusively under anaesthesia in the theatre. This makes procedural sedation and analgesia invaluable. Ketamine has been proven to be safe and effective, and is relatively cheap.^41,42^ For these reasons, anaesthesia and conscious sedation for painful procedures in resource-limited settings, particularly for children, remains largely ketamine-based.^33^ The expert panel agreed that ketamine is essential as the cornerstone of procedural analgesia for the analgesia protocol. There was also consensus regarding top-up doses to overcome tachyphylaxis.

## Limitations

A selection of specialists is required for the participant panel for the Delphi technique, by virtue of the design and method. The number of doctors with the required expertise to participate was low. Although 10 healthcare professionals were identified to participate, only nine agreed to participate in round one and all nine panellists were not retained in round two. The small number of panellists renders the results not generalisable. All of the panellists included were males. There are very few female surgeons managing burns in Africa. As a result, there were no females in our panel. Whilst we do not feel this would have influenced our results, it would have been beneficial to have included female panellists.

## Conclusion

A modified Delphi method was used to obtain expert consensus for a recently adopted analgesia protocol for burn-injured children in a resource-limited setting, with experts in the management of burn injuries in low and middle-income settings. The expert consensus leads to the rigour and robustness of the protocol. Delphi methods are exceptionally valuable in healthcare research and the aim of such studies is to find converging expert opinions.
